# The Relationship Between Mental Health and Periodontal Disease: Insights from NHANES Data

**DOI:** 10.12688/f1000research.150837.1

**Published:** 2024-06-27

**Authors:** Eman AlJoghaiman

**Affiliations:** 1 Department of Preventive Dental Science, College of Dentistry, Imam Abdulrahman Bin Faisal University, Dammam, Eastern Province, 12372, Saudi Arabia

**Keywords:** Stress; mental health; periodontal disease; NHANES

## Abstract

**Introduction and aim:**

Periodontal disease, initiated by dental biofilm and influenced by various local and systemic factors, includes stress as a potential contributor to its progression. Despite associations with severe forms like acute necrotizing ulcerative gingivitis, a comprehensive large-sample study linking stress to periodontal disease is lacking. This study aims to investigate the relationship between mental health and periodontal disease.

**Materials and Methods:**

Leveraging data from the National Health and Nutrition Examination Survey (NHANES), relevant information was extracted. Mental health was the exposure variable, and periodontal disease, assessed through indices following Eke et al.'s definition, served as the outcome. Covariates impacting periodontal disease were considered, and demographic and disease status analyses employed the Rao-Scott chi-squared test. A logistic regression model assessed mental health's impact on periodontal disease.

**Results:**

Logistic regression indicated higher odds of periodontal disease among individuals feeling bad about themselves for more than half of the day (OR 1.170, 95% CI 0.533-2.474), though statistical significance was not reached. Periodontitis prevalence significantly varied based on marital status, with 6.6% of married and 10.8% of unmarried subjects affected. Notably, a statistically significant difference in periodontitis prevalence existed between subjects with health insurance (8.3%) and those without (16.5%).

**Conclusion:**

Within study limitations, it is concluded that no significant difference exists in periodontal status between individuals with compromised mental health and those without. The findings underscore potential associations between mental health, marital status, and access to health insurance with periodontal disease.

## Introduction

Periodontal disease is a multifaceted condition influenced by various factors. Its distinctive characteristics, site-specific progression involving complex etiological factors, and the impact of risk factors have continually spurred researchers to delve deeper into unraveling the intricacies of the disease process. ^1^ While the disease typically begins with dental biofilm, transitioning from gingivitis to periodontitis, it's important to emphasize that not all instances of gingivitis evolve into periodontitis. Furthermore, there's notable variation in the prevalence of periodontitis within a given population, with some individuals showing no signs, others exhibiting slow progression, and some experiencing a more rapid advancement. ^2^ Additionally, on an individual level, not every site displays clinical attachment loss and signs of periodontal disease; certain sites may present severe clinical loss of attachment coupled with bone loss. ^3^

The diverse manifestations of periodontal disease may arise due to the intricate nature of factors influencing its progression. Both local and systemic factors can exert an influence on the development of the disease. ^4^ Numerous systemic factors that contribute to the progression of the disease have been thoroughly elucidated. These encompass diabetes, hormonal fluctuations during puberty, pregnancy, and menopause, as well as genetic factors and conditions. ^5^ Stress and psychological factors are also recognized contributors, impacting the overall periodontal health status and influencing disease progression. ^6^

Psychological stress refers to the emotional and physiological response individuals undergo when confronted with challenging life events. These events may include exams, adapting to new work conditions, marital conflicts, financial instability, or even profound situations like the loss of a loved one. The intensity of these situations surpasses an individual's ability to cope effectively, triggering emotional and physiological reactions. It is a significant modifiable risk factor impacting both mental and physical well-being. ^7^^,^^8^

The term “stress” encompasses any physical or psychological event perceived as capable of causing harm or emotional distress. Stress is a ubiquitous factor in nearly all chronic diseases. Psychosocial stress is subject to modification by individual perceptions and coping strategies, involving the release of specific products through various pathways: the hypothalamic-pituitary-adrenal axis, leading to glucocorticosteroids; the autonomic nervous system, resulting in the release of catecholamines; and the hypothalamic-pituitary-gonadal axis, leading to the release of sex hormones. ^9^ Exposure to stress during critical periods is known to alter hormonal and immune systems. The emotional or psychological burden may directly influence immune activities through nerve messenger substances or indirectly through hormones. The oral cavity, as the gateway for systemic components, is not immune to the effects of stress. ^10^

In the pathophysiological models proposed by Genco et al. (1998), the role of stress as a risk factor for periodontal disease is thoroughly explained. Two models are presented, one detailing the direct effects and the other describing the indirect effects. The primary model posits that psychosocial stressors set off a sequence of events involving the release of corticotrophin-releasing hormone through the hypothalamic-pituitary-adrenal axis, the autonomic nervous system, and the central nervous system. These physiological responses negatively impact the immune-inflammatory cells, increasing the susceptibility to infection and, specifically, periodontal disease. The second model outlines the indirect impact of stress on health-risk behaviors such as inadequate oral hygiene, smoking, overeating, and particularly a high-fat diet, which can result in immunosuppression due to heightened cortisol production. Another potential behavioral consequence influenced by stress and inadequate coping is depression. The interplay of all these factors contributes to the progression of periodontal disease. ^9^^,^^11^

The connection between stress and periodontal disease has long been established. Stress is linked to acute necrotizing ulcerative gingivitis, with immune-compromised conditions and poor oral hygiene as contributing factors for this acute condition. ^12^ Stress and depression can compromise periodontal resistance, fostering opportunistic microbial growth, reducing saliva flow, and impairing gingival blood flow. ^13^ Elevated levels of epinephrine and non-epinephrine induced by stress alter blood flow and oxygen requirements. Changes in the host response, particularly in neutrophil function, create an environment conducive to bacterial growth, including species like Prevotella intermedia and other spirochetes, leading to necrotizing ulcerative gingivitis. ^14^ This pathogenic process is supported by increased clinical cases observed during stressful periods, such as exams in students and in individuals with demanding occupations. ^15^ The literature also reflects a similar correlation between stress and severe periodontitis. ^16^ Furthermore, in individuals experiencing stress, the virus is believed to reside in connective tissue, contributing to periodontal disease progression alongside other microbial complexities. ^17^

While studies spanning four decades exist, the number of investigations correlating stress and periodontal disease remains limited. The challenge lies in the varied definitions used to measure stress, encompassing different types of stress, psychological disorders, and various subcategories. These complexities make it challenging to clearly understand the link between stress and periodontal disease. Various types of psychological and psychosocial factors, such as high work stress, job dissatisfaction or unemployment, family status, and the impact of major life events, have been studied and found to be related to periodontal disease. ^18^^–^^20^ However, none of these studies distinctly define this association.

In a study by Genco et al. (1999), psychosocial stress factors, coupled with financial worries, were associated with adult periodontal disease, highlighting both as risk indicators. ^21^ Another study correlating patients with inadequate stress behavior strategies (defensive coping) with periodontal disease identified these individuals as being at a higher risk for severe periodontal disease. However, the necessity for further investigations was emphasized to elucidate their role in the progression of periodontal disease. ^22^

Occupational stress emerges as a potential risk factor for periodontal disease, as evidenced by a study conducted among police personnel. The experimental group, characterized by higher occupational index scores and serum cortisol levels, exhibited elevated plaque scores and bleeding indices compared to the control group. ^23^ Another study by Vyas (2018) explored the correlation between stress, measured by cortisol levels, in two groups of 25 patients each with chronic periodontitis and a healthy periodontium. Significant differences were observed in plaque, probing depth, clinical attachment level, and cortisol levels, leading to the conclusion that psychosocial stress may be associated with periodontal destruction through both behavioral and physiological mechanisms. ^24^ Despite these findings highlighting the impact of stress on periodontal disease, the direct effects remain somewhat unclear. Furthermore, existing studies underscore the need for further research to establish this impact on a larger population, considering other controlled variables. Consequently, the present study seeks to investigate the relationship between mental health (self-perception) and periodontal disease.

## Methods

### Study design and population ^25^

The National Health and Nutrition Examination Survey (NHANES) is a comprehensive cross-sectional survey conducted in the United States, designed to offer a nationally representative overview of non-institutionalized individuals residing in households. Participants in this survey undergo a series of assessments, which include completing a questionnaire, undergoing medical and dental examinations, and participating in various laboratory tests. The protocols for collecting oral health data in the NHANES 2011–2012 and NHANES 2013–2014 cycles received approval from the Centers for Disease Control and Prevention National Center for Health Statistics Research Ethics Review Board. All survey participants provided written informed consent before publishing their information. Our study utilized data from key sections of the NHANES, encompassing demographic information, examination results, questionnaire responses, and limited access data. Specifically focusing on individuals aged 18 years and older who underwent a dental examination, we implemented exclusion criteria to exclude edentulous subjects from our analysis. This strategy ensures a thorough and targeted evaluation of oral health within a diverse and nationally representative sample of the U.S. population.

### Variable of interest

**Exposure variable:**

Mental health

**Outcome variable:**

Periodontal disease

For this investigation, we used the NHANES complete periodontal examination data to calculate periodontal disease indices using definition of periodontal disease (1). According to the definition, periodontal disease was classified as follows: Severe periodontitis: >=2 interproximal sites with loss of attachment (LOA) >=6 mm (not on the same tooth) and >=1 interproximal site with probing depth (PD) >=5 mm; Moderate periodontitis: >=2 interproximal sites with LOA >=4 mm (not on same tooth), or >=2 interproximal sites with PD>=5 mm (not on same tooth); Mild periodontitis: >=2 interproximal sites with LOA >=3 mm, and >=2 interproximal sites with PD >=4 mm (not on same tooth) or one site with PD >=5 mm and finally, no periodontitis group whose has no evidence of mild, moderate, or severe periodontitis. ^26^

**Covariate variable:**

To ensure a comprehensive examination and control for potential influencing factors, our analysis incorporates a diverse set of covariates. These covariates play a crucial role in minimizing the impact of potential confounders, enabling a more precise scrutiny of the relationship between the exposure and outcome. The extensive list of covariates comprises age, sex, race/ethnicity, education, socioeconomic status, poverty/income ratio, marital status, occupation, smoking habits, alcohol consumption, BMI, HbA1C, dental insurance coverage, dental visit frequency, and for mental health assessment.

Age is divided into six groups: (18-30), (31-40), (41-50), (51-60), and over 60 years. Gender is categorized as either female or male. Race and ethnicity are classified as non-Hispanic White, non-Hispanic Black, Mexican American and other Hispanic, and non-Hispanic Asian. Poverty indices are categorized into low, middle, and high. Marital status is indicated as yes or no. Occupation is categorized as working and non-working. Dental visits are classified as regular and not regular. Dental insurance coverage is delineated as yes or no. Smoking status is divided into never, former, and current smokers. Alcohol consumption is classified as alcohol drinker and non-alcohol drinker.

By meticulously examining and accounting for these covariates, our objective is to obtain results that reflect the true relationship between the exposure and outcome, minimizing the confounding effects of other variables.

### Statistical analysis

The data were derived by consolidating information from demographic records, health questionnaires, clinical examinations, and limited access data sourced from NHANES (2011–2012) and corresponding files from NHANES (2013–2014). To ensure unbiased point estimates and accurate variance estimation, given the complex sampling design of NHANES, appropriate sampling weights were applied. SAS version 9.4, survey procedures were utilized, following the guidelines set by the National Center for Health Statistics and the Centers for Disease Control and Prevention.

To analyze the demographics and disease status of the study population, the Rao-Scott chi-squared test was employed, taking into consideration the survey design complexities. Additionally, a logistic regression model was utilized to assess the influence of mental health on periodontal disease. The significance level was established at p ≤ 0.05, ensuring a stringent evaluation of the relationships within the study. These methodological approaches contribute to the robustness and reliability of our findings.

## Results

### Study characteristic

The demographic characteristics of the study subjects are presented in Table 1, with weighted percentages. Among the 2764 subjects, more than a quarter (29.1%) were aged over 60 years, 52% were females, 42.1% identified as non-Hispanic white, 38.7% reported a high household level, and over half (54.1%) held either an associate or college degree. A majority of subjects had some form of health insurance, and the majority had visited the dentist within the 12 months preceding the survey.

**Table 1.** Demographic characteristics of the study subjects (n=2764).

	Total (n)	Percentage % ^a^
**Age**		
18-30 years	628	22.7
31-40 years	428	15.5
41-50 years	452	16.4
51-60 years	453	16.4
More than 60 years	803	29.1
**Gender**		
Male	1328	48.0
Female	1436	52.0
**Race/ethnicity**		
Mexican American	378	13.7
Other Hispanic	246	8.9
Non-Hispanic White	1165	42.1
Non-Hispanic Black	566	20.5
Other Races Including Multi-Racial	409	14.8
**Household income**		
Below Poverty Line	843	30.5
Near Poverty	112	4.1
Low-Income	419	15.2
Middle-Income	320	11.6
High-Income	1070	38.7
**Education level**		
Less than High School	568	20.5
High School Level	620	22.4
AA or College Degree	1495	54.1
**Weight status (Based on BMI)**		
Underweight	770	27.9
Normal	738	26.7
Overweight	584	21.1
Obese	672	24.3
**Alcohol consumption status**		
Non-drinker	1770	64.0
Drinker	994	36.0
**Diabetes**		
Present	277	10.0
Not present	2487	90.0
**Periodontal disease**		
Severe	3	0.1
Moderate	28	1.0
Mild	241	8.7
Not present	2492	90.2
**Time of most recent dental visit**		
Less than 1 year	1741	63
1-2 years	498	18
More than 2 years	525	19
**Insurance coverage**		
Yes	2255	82
No	509	18
**Mental health**		
Not at all	2316	83.8
Several days	288	10.4
More than half the days	70	2.5
Nearly every day	90	3.3

^a^ Weighted row percentages.

### Effect of sociodemographic factors on the prevalence of periodontitis

The subjects were categorized based on the severity of periodontitis, as outlined in Tables 2 and 3, distinguishing between no, mild, moderate, and severe cases. Significant differences in the prevalence of periodontitis were observed based on subjects' education levels. However, no significant differences were noted in the prevalence of periodontitis based on the subjects' mental health. Notably, the prevalence of periodontitis varied significantly according to subjects' marital status, with approximately 6.6% of married subjects and 10.8% of unmarried subjects experiencing some form of periodontitis. A statistically significant association was found.

**Table 2.** Subject demographics and demographic predictors of periodontics in study subjects (n=2764).

	Total (n)	Severe periodontitis %	Moderate periodontitis %	Mild periodontitis %	No periodontitis %	*P*-value *
**Age**						
18-30	628	0.0%	0.5%	7.6%	91.9%	0.353
31-40	428	0.0%	1.4%	8.9%	89.7%	
41-50	452	0.0%	0.4%	10.2%	89.4%	
51-60	453	0.0%	1.1%	7.7%	91.2%	
More than 60 years	803	0.4%	1.5%	9.2%	88.9%	
**Gender**						
Male	1328	0.2%	0.7%	9.3%	89.8%	0.269
Female	1436	0.0%	1.3%	8.1%	90.5%	
**Race/ethnicity**						
Mexican American	378	0.0%	1.1%	7.7%	91.3%	0.587
Other Hispanic	246	0.0%	0.4%	8.5%	91.1%	
Non-Hispanic White	1165	0.2%	1.4%	7.9%	90.6%	
Non-Hispanic Black	566	0.2%	0.5%	9.7%	89.6%	
Other Races Including Multi-Racial	409	0.0%	1.0%	10.8%	88.3%	
**Household income**						
Below Poverty Line	843	0.1%	1.2%	8.8%	89.9%	0.822
Near Poverty	112	0.0%	1.8%	8.0%	90.2%	
Low-Income	419	0.0%	1.0%	7.6%	91.4%	
Middle-Income	320	0.3%	0.9%	10.0%	88.8%	
High-Income	1070	0.1%	0.8%	8.8%	90.3%	
**Education level**						
Less than High School	568	0.0%	0.7%	6.5%	92.8%	0.050 *
High School Level	620	0.2%	1.3%	10.0%	88.5%	
AA or College Degree	1495	0.1%	1.0%	8.8%	90.0%	
**Mental health**						
Not at all	2316	0.1%	0.9%	8.9%	90.1%	0.686
Several days	288	0.0%	2.4%	6.9%	90.6%	
More than half the days	70	0.0%	0.0%	11.4%	88.6%	
Nearly every day	90	0.0%	0.0%	7.9%	92.1%	
**Occupation**						
Working	1470	0.0%	1.4%	8.2%	0.0%	0.343
Not Working	1294	0.2%	0.6%	9.3%	0.2%	
**Marital status**						
Married	2157	0.00%	0.00%	6.60%	93.40%	0.001 *
Not Married	607	0.10%	1.30%	9.30%	89.20%	
**Alcohol consumption status**						
Non-drinker	1770	0.2%	1.1%	8.3%	90.4%	0.311
Drinker	994	0.0%	0.8%	9.5%	89.7%	
**Weight status (Based on BMI)**						
Underweight	0.0%	1.0%	7.7%	91.3%	0.0%	0.524
Normal	0.0%	0.9%	8.7%	90.4%	0.0%	
Overweight	0.3%	1.4%	9.2%	89.0%	0.3%	
Obese	0.1%	0.7%	9.5%	89.6%	0.1%	
**Diabetes**						
Present	277	0.7%	0.7%	8.3%	90.3%	0.529
Not present	2487	0.0%	1.0%	8.8%	90.1%	

^a^ Weighted row percentages.

* By Rao-Scott chi-square test.

**Table 3.** Distribution of Periodontitis per Dental Visits, Insurance Coverage, Occupation, and, Marital Status Among Study Subjects (n=2764).

	Total (n)	Severe periodontitis %	Moderate periodontitis %	Mild periodontitis%	No periodontitis %	*P*-value ^a^
**Time of most recent dental visit**						
Less than 1 year	1754	0.1%	1.2%	8.6%	90.1%	0.998
1-2 years	274	0.4%	0.4%	9.1%	90.1%	
More than 2 years	736	0.1%	0.8%	8.8%	90.2%	
**Health Insurance**						
Yes	2255	0.1%	0.9%	7.3%	91.7%	0.001 ^a^
No	509	0.0%	1.4%	15.1%	83.5%	

^a^ Weighted row percentages.

Unadjusted odds ratios (OR) were computed through logistic regression, controlling for all confounders, and are presented with a 95% confidence interval (CI) in Table 4. The logistic regression analysis indicated that the odds of periodontal disease were higher among those who reported feeling bad about themselves for more than half of the day compared to those who reported not feeling that way at all (OR 1.170, 95% CI 0.533-2.474). However, this association was not statistically significant (p>0.05).

**Table 4.** Logistic regression analysis of the effects of mental health on periodontitis.

Variable	OR (95% CI)
**Mental health**	
Not at all	Ref
Several days	0.938 (0.617-1.426)
More than half the days	1.170 (0.533-2.473)
Nearly every day	0.765 (0.349-1.674)

No Periodontitis=0 vs. Periodontitis (all three)=1.

## Discussion

The primary objective of this study was to examine the relationship between mental health (self-perception) and periodontal disease. The study findings revealed no discernible difference in the prevalence of periodontitis between individuals with mental health issues and those without. However, it is noteworthy that the odds of periodontal disease were somewhat higher among those who reported feeling bad about themselves for more than half of the day, though this difference did not reach statistical significance. Additionally, male participants exhibited a higher likelihood of periodontal disease compared to female participants. Furthermore, lacking insurance coverage was significantly associated with the prevalence of periodontitis.

The study findings presented here cannot be directly compared with other studies to date due to variations in the measures used to assess stress and psychosocial conditions. Nevertheless, common factors across these studies include stress as a factor and the outcome variable, which is periodontal disease.

Monteiro da Silva et al. (1998) found no significant difference between psychosocial stress and periodontal disease. ^27^ Similarly, Solis et al. (2004) reported no association between depression, hopelessness, psychiatric symptoms, and established or severe periodontitis. ^28^ Castro et al. (2006) also concluded that there was no significant association between periodontitis and the analyzed psychosocial factors. ^29^ The present study aligns with the findings of Shende et al. (2016), where the oral hygiene and periodontal status of individuals with mild anxiety and depression were not different from those without anxiety and depression. ^30^ Thus, this study does not demonstrate stress as a causative factor for periodontal disease, despite observing a difference in the periodontal status of individuals with or without normal mental health, which was not statistically significant.

The current study's results are consistent with Genco et al.'s (1998) findings, indicating that various measures of stress, including life events and daily strains, were not correlated with periodontal disease. Only financial strain showed a significant association with greater attachment loss and alveolar bone loss after adjusting for variables such as age and smoking. Subjects with higher financial strain and those with depression had a significantly higher risk for periodontal disease. ^11^

One intriguing observation in the present study was the lower prevalence of periodontal disease and related findings in individuals with dental insurance compared to those without insurance. The possession of dental insurance appears to contribute to a sense of mental well-being, potentially reducing financial-related stress and mitigating the negative impact on periodontal disease. This aligns with the findings of Genco et al. (1998), who demonstrated a correlation between financial strain and poor periodontal health. ^11^ Similarly, a study exploring the relationship between mental health and oral health in older individuals in Australia highlighted that the availability of free treatment or provision of dental insurance offers a sense of comfort in daily life, potentially shielding against mental health issues. ^31^ Ng and Leung (2006) also support this perspective, suggesting that individuals with higher mean clinical attachment level values tended to have higher scores on job and financial strain scales compared to periodontally healthy individuals. ^32^

The findings of the present study diverge from those reported by Mahendra et al. (2011), Reshma et al. (2013), Vyas et al. (2018), and various other animal and human studies. ^23^^,^^24^^,^^33^ These studies employed diverse stress measures, including cortisol levels and other stress assessment methods. Discrepancies between the present study's findings and those of the aforementioned studies could arise from differences in methodology, the type of assessment employed, considered co-variables, and the demographic characteristics of the study participants. Many studies focused on specific groups exposed to stress, such as police personnel or older individuals, whereas our study encompassed a broader sample, considering individuals from various backgrounds in relation to mental health. The diverse nature of the study population may contribute to the variations observed in the results.

The current investigation has notable strengths, including its reliance on data from a large-scale population-based survey with a robust sampling and weighting system. This survey encompassed a diverse range of participants, including both females and males, various age groups, individuals with different socioeconomic statuses, and those with and without insurance. The inclusion of these variables, which are not often considered collectively in previous studies, enhances the reliability of the associations found within this research. However, there are limitations to consider. Firstly, mental health status was assessed based on self-reported answers from the participants, which may introduce subjectivity and potential bias into the study outcomes. The broad and complex nature of assessing mental health on a single-point measure or limited parameters may not fully capture the true mental health status of individuals. Additionally, the cross-sectional design of the study limits the ability to infer causality in the observed associations. The study does not confirm the outcome, and the link between stress-associated odds of periodontal disease and behavioral or pathophysiological changes remains to be determined.

Moreover, the study acknowledges the potential bidirectional relationship between oral health concerns and mental health status. For instance, the appearance of teeth, mouth, or dentures, as well as problems with them, might impact social interaction, potentially contributing to mental health issues such as feelings of hopelessness and depression. Controlling for these variables in a study investigating the relationship between mental health status and periodontal disease becomes challenging, and the true measured outcome may be affected.

To enhance future research, prospective studies with a focus on biochemical and physiological mechanisms by which psychosocial stress contributes to periodontal destruction are needed. These studies could establish the biological rationale for the observed relationship. Furthermore, refining the definition of stress and employing a standardized protocol to assess stress and associated factors would contribute to clearer comparisons of periodontal status among individuals.

## Conclusion

In conclusion, within the constraints of this study, it can be deduced that there is no statistically significant difference in the periodontal status between individuals with compromised mental health and those without such issues. The findings suggest that mental health, as assessed through self-reported responses, may not be a robust predictor of periodontal disease in the studied population. Despite observing higher odds of periodontal disease among individuals who reported feeling bad about themselves for more than half of the day, this difference did not reach statistical significance.

## Ethics and consent

The protocols for collecting oral health data in the NHANES 2011–2012 and NHANES 2013–2014 cycles received approval from the Centers for Disease Control and Prevention National Center for Health Statistics Research Ethics Review Board. All survey participants provided written informed consent before publishing their information. https://www.cdc.gov/nchs/nhanes/irba98.htm

## Data Availability

Aljoghaiman, Eman (2024). The Relationship Between Mental Health and Periodontal Disease: Insights from NHANES Data. figshare. Dataset. https://doi.org/10.6084/m9.figshare.25662735 ^34^ Data are available under the terms of the Creative Commons Attribution 4.0 International license (CC-BY 4.0)
