# The Relationship Between Mental Health and Periodontal Disease: Insights from NHANES Data

**DOI:** 10.12688/f1000research.150837.3

**Published:** 2025-10-31

**Authors:** Eman AlJoghaiman

**Affiliations:** 1 Department of Preventive Dental Science, College of Dentistry, Imam Abdulrahman Bin Faisal University, Dammam, Eastern Province, 12372, Saudi Arabia

**Keywords:** Stress; mental health; periodontal disease; NHANES

## Abstract

**Introduction and aim:**

Periodontal disease, initiated by dental biofilm and influenced by various local and systemic factors, includes stress as a potential contributor to its progression. Despite associations with severe forms like acute necrotizing ulcerative gingivitis, a comprehensive large-sample study linking stress to periodontal disease is lacking. This study aims to investigate the relationship between mental health and periodontal disease.

**Materials and Methods:**

Leveraging data (secondary dataset) from the National Health and Nutrition Examination Survey (NHANES) from 2011–2012 and NHANES 2013–2014 cycles, relevant information was extracted. Mental health was the exposure variable, and periodontal disease, assessed through indices following Eke et al.’s definition, served as the outcome. Covariates (demographical characteristics) impacting periodontal disease were considered, and disease status analyses employed the Rao-Scott chi-squared test. A logistic regression model assessed mental health’s impact on periodontal disease.

**Results:**

Among the 2764 Participants, more than a quarter (29.1%) were aged over 60 years, 52% were females. Logistic regression indicated higher odds of periodontal disease among individuals feeling bad about themselves for more than half of the day (OR 1.170, 95% CI 0.533-2.474), though statistical significance was not reached. Periodontitis prevalence significantly varied based on marital status, with 6.6% of married and 10.8% of unmarried Participants affected. Notably, a statistically significant difference in periodontitis prevalence existed between Participants with health insurance (8.3%) and those without (16.5%).

**Conclusion:**

Our findings suggest trends in periodontal disease prevalence linked to mental health, marital status, and access to health insurance. However, the absence of statistically significant findings calls for caution in interpreting these relationships. We recommend that future studies further investigate these potential associations to provide a clearer understanding.

## Introduction

Periodontal disease is a multifaceted condition influenced by various factors. Its distinctive characteristics, site-specific progression involving complex etiological factors, and the impact of risk factors have continually spurred researchers to delve deeper into unraveling the intricacies of the disease process. ^1^ While the disease typically begins with dental biofilm, transitioning from gingivitis to periodontitis, it’s important to emphasize that not all instances of gingivitis evolve into periodontitis. Furthermore, there’s notable variation in the prevalence of periodontitis within a given population, with some individuals showing no signs, others exhibiting slow progression, and some experiencing a more rapid advancement. ^2^ Additionally, on an individual level, not every site displays clinical attachment loss and signs of periodontal disease; certain sites may present severe clinical loss of attachment coupled with bone loss. ^3^

The diverse manifestations of periodontal disease may arise due to the intricate nature of factors influencing its progression. Both local and systemic factors can exert an influence on the development of the disease. ^4^ Numerous systemic factors that contribute to the progression of the disease have been thoroughly elucidated. These encompass diabetes, hormonal fluctuations during puberty, pregnancy, and menopause, as well as genetic factors and conditions. ^5^ Stress and psychological factors are also recognized contributors, impacting the overall periodontal health status and influencing disease progression. ^6^

Psychological stress refers to the emotional and physiological response individuals undergo when confronted with challenging life events. These events may include exams, adapting to new work conditions, marital conflicts, financial instability, or even profound situations like the loss of a loved one. The intensity of these situations surpasses an individual’s ability to cope effectively, triggering emotional and physiological reactions. It is a significant modifiable risk factor impacting both mental and physical well-being. ^7^^,^^8^

The term “stress” encompasses any physical or psychological event perceived as capable of causing harm or emotional distress. Stress is a ubiquitous factor in nearly all chronic diseases. Psychosocial stress is subject to modification by individual perceptions and coping strategies, involving the release of specific products through various pathways: the hypothalamic-pituitary-adrenal axis, leading to glucocorticosteroids; the autonomic nervous system, resulting in the release of catecholamines; and the hypothalamic-pituitary-gonadal axis, leading to the release of sex hormones. ^9^ Exposure to stress during critical periods is known to alter hormonal and immune systems. The emotional or psychological burden may directly influence immune activities through nerve messenger substances or indirectly through hormones. The oral cavity, as the gateway for systemic components, is not immune to the effects of stress. ^10^

In the pathophysiological models proposed by Genco et al. (1998), the role of stress as a risk factor for periodontal disease is thoroughly explained. Two models are presented, one detailing the direct effects and the other describing the indirect effects. The primary model posits that psychosocial stressors set off a sequence of events involving the release of corticotrophin-releasing hormone through the hypothalamic-pituitary-adrenal axis, the autonomic nervous system, and the central nervous system. These physiological responses negatively impact the immune-inflammatory cells, increasing the susceptibility to infection and, specifically, periodontal disease. The second model outlines the indirect impact of stress on health-risk behaviors such as inadequate oral hygiene, smoking, overeating, and particularly a high-fat diet, which can result in immunosuppression due to heightened cortisol production. Another potential behavioral consequence influenced by stress and inadequate coping is depression. The interplay of all these factors contributes to the progression of periodontal disease. ^9^^,^^11^

The connection between stress and periodontal disease has long been established. Stress is linked to acute necrotizing ulcerative gingivitis, with immune-compromised conditions and poor oral hygiene as contributing factors for this acute condition. ^12^ Stress and depression can compromise periodontal resistance, fostering opportunistic microbial growth, reducing saliva flow, and impairing gingival blood flow. ^13^ Elevated levels of epinephrine and non-epinephrine induced by stress alter blood flow and oxygen requirements. Changes in the host response, particularly in neutrophil function, create an environment conducive to bacterial growth, including species like Prevotella intermedia and other spirochetes, leading to necrotizing ulcerative gingivitis. ^14^ This pathogenic process is supported by increased clinical cases observed during stressful periods, such as exams in students and in individuals with demanding occupations. ^15^ The literature also reflects a similar correlation between stress and severe periodontitis. ^16^ Furthermore, in individuals experiencing stress, the virus is believed to reside in connective tissue, contributing to periodontal disease progression alongside other microbial complexities. ^17^

While studies spanning four decades exist, the number of investigations correlating stress and periodontal disease remains limited. The challenge lies in the varied definitions used to measure stress, encompassing different types of stress, psychological disorders, and various subcategories. These complexities make it challenging to clearly understand the link between stress and periodontal disease. Various types of psychological and psychosocial factors, such as high work stress, job dissatisfaction or unemployment, family status, and the impact of major life events, have been studied and found to be related to periodontal disease. ^18^^–^^20^ However, none of these studies distinctly define this association.

Self-perception can be viewed as a form of psychological and psychosocial stress that significantly influences overall mental health. Negative self-perception often leads to heightened stress levels, resulting in anxiety and depressive symptoms. ^21^ Individuals who struggle with low self-esteem frequently find it challenging to cope with life’s difficulties, which can further exacerbate their mental health issues. ^22^ This psychological distress not only affects their emotional well-being but can also manifest in maladaptive behaviors, further compromising their health.

The relationship between self-perception and periodontal disease is an area of growing concern. Individuals with poor self-image and elevated stress levels may neglect their oral hygiene, increasing their susceptibility to periodontal conditions. ^23^ Moreover, the stress associated with negative self-perception can lead to inflammatory responses that adversely affect periodontal health. ^24^ Despite these associations, there is a notable gap in the literature regarding the comprehensive understanding of how psychological factors like self-perception influence periodontal disease. Therefore, our study investigates the association between mental health problems, specifically self-perception, and periodontal disease to address this gap and contribute to the existing body of knowledge.

## Methods

### Study design and population ^25^

This cross-sectional analytical study utilized publicly available data from the National Health and Nutrition Examination Survey (NHANES) 2011–2012 and 2013–2014 cycles. NHANES employs a complex, multistage probability sampling strategy to obtain a nationally representative sample of non-institutionalized U.S. adults. Participants complete structured household interviews and undergo standardized clinical examinations in mobile units operated by trained personnel.

### Study population

Adults aged ≥18 years who completed the periodontal examination and mental health questionnaire were eligible. Participants were excluded if they were edentulous, lacked periodontal assessments, or had incomplete data for the primary exposure or outcome variables. NHANES sample weights were applied to account for differential probabilities of selection and to yield nationally representative estimates.

### Variable of interest

**Periodontal examination and outcome definition**

Periodontal status was assessed using clinical attachment loss (CAL) and probing depth (PD) measurements collected at six sites per tooth. Periodontitis case status was defined using the Centers for Disease Control and Prevention/American Academy of Periodontology (CDC/AAP) case definitions, which classify disease based on specified thresholds of interproximal CAL and PD. ^26^

**Mental health exposure**

Psychological distress was the primary exposure variable. Participants were asked how often during the previous two weeks they had been bothered by feeling bad about themselves or feeling like a failure. Responses were recorded on a four-point scale: *Not at all, Several days, More than half the days,* and *Nearly every day*. For analysis, this variable was retained in its original ordinal structure to reflect severity gradients of negative self-perception.

**Covariate variable**

Covariates were selected according to prior literature establishing links with periodontal outcomes. Sociodemographic variables included age (categorized into NHANES adult age groups), sex, education, and socioeconomic status, measured using the income-to-poverty ratio. Health-related variables included body mass index (BMI), categorized as underweight (<18.5 kg/m ^2^), normal weight (18.5–24.9 kg/m ^2^), overweight (25.0–29.9 kg/m ^2^), or obese (≥30.0 kg/m ^2^), and self-reported physician-diagnosed diabetes. Alcohol consumption was classified as current drinkers versus non-drinkers. Dental care utilization was determined by time since last dental visit (≤ *1 year, 1–2 years,* and > *2 years*), and dental insurance coverage was categorized as insured versus uninsured. All covariates were obtained from standardized NHANES interview modules.

### Statistical analysis

Analyses accounted for NHANES sampling weights, strata, and primary sampling units to ensure nationally representative variance estimation. Descriptive statistics summarized the distribution of study variables. Bivariate comparisons between psychological distress categories and periodontitis were evaluated using chi-square tests.

Survey-weighted multivariable logistic regression models were constructed to estimate adjusted odds ratios (ORs) and 95% confidence intervals (CIs) for the association between psychological distress and periodontitis, controlling for covariates. A significance level of **α = 0.05** was set for all statistical tests. Data analysis was performed using SAS version 9.4 (SAS Institute Inc., Cary, NC).

## Results

### Study characteristic

The demographic characteristics of the study Participants are presented in Table 1. Among the 2764 Participants, more than a quarter (29.1%) were aged over 60 years, (52%) were females, (42.1%) identified as non-Hispanic white, (38.7%) reported a high household level, and over half (54.1%) held either an associate or college degree. A majority of Participants had some form of health insurance, and the majority had visited the dentist within the 12 months preceding the survey.

**Table 1.** Demographic characteristics of the study Participants (n=2764).

	Total (n)	Percentage % ^a^
**Age**		
18-30 years	628	22.7
31-40 years	428	15.5
41-50 years	452	16.4
51-60 years	453	16.4
More than 60 years	803	29.1
**Gender**		
Male	1328	48.0
Female	1436	52.0
**Race/ethnicity**		
Mexican American	378	13.7
Other Hispanic	246	8.9
Non-Hispanic White	1165	42.1
Non-Hispanic Black	566	20.5
Other Races Including Multi-Racial	409	14.8
**Household income**		
Below Poverty Line	843	30.5
Near Poverty	112	4.1
Low-Income	419	15.2
Middle-Income	320	11.6
High-Income	1070	38.7
**Education level**		
Less than High School	568	20.5
High School Level	620	22.4
AA or College Degree	1495	54.1
**Weight status (Based on BMI)**		
Underweight	770	27.9
Normal	738	26.7
Overweight	584	21.1
Obese	672	24.3
**Alcohol consumption status**		
Non-drinker	1770	64.0
Drinker	994	36.0
**Diabetes**		
Present	277	10.0
Not present	2487	90.0
**Periodontal disease**		
Severe	3	0.1
Moderate	28	1.0
Mild	241	8.7
Not present	2492	90.2
**Time of most recent dental visit**		
Less than 1 year	1741	63
1-2 years	498	18
More than 2 years	525	19
**Insurance coverage**		
Yes	2255	82
No	509	18
**Mental health**		
Not at all	2316	83.8
Several days	288	10.4
More than half the days	70	2.5
Nearly every day	90	3.3

^a^ Weighted row percentage.

### Association of sociodemographic factors on the prevalence of periodontitis

The Participants were categorized based on the severity of periodontitis, as outlined in Tables 2 and 3, distinguishing between no, mild, moderate, and severe cases. Significant differences in the prevalence of periodontitis were observed based on Participants’ education levels. However, no significant differences were noted in the prevalence of periodontitis based on the Participants’ mental health. Notably, the prevalence of periodontitis varied significantly according to Participants’ marital status, with approximately (6.6%) of married Participants and (10.8%) of unmarried Participants experiencing some form of periodontitis. A statistically significant association was found.

**Table 2.** Subject demographics and demographic predictors of periodontics in study Participants (n=2764).

	Total (n)	Severe periodontitis %	Moderate periodontitis %	Mild periodontitis %	No periodontitis %	*P*-value
**Age**						
18-30	628	0.0%	0.5%	7.6%	91.9%	0.353
31-40	428	0.0%	1.4%	8.9%	89.7%	
41-50	452	0.0%	0.4%	10.2%	89.4%	
51-60	453	0.0%	1.1%	7.7%	91.2%	
More than 60 years	803	0.4%	1.5%	9.2%	88.9%	
**Gender**						
Male	1328	0.2%	0.7%	9.3%	89.8%	0.269
Female	1436	0.0%	1.3%	8.1%	90.5%	
**Race/ethnicity**						
Mexican American	378	0.0%	1.1%	7.7%	91.3%	0.587
Other Hispanic	246	0.0%	0.4%	8.5%	91.1%	
Non-Hispanic White	1165	0.2%	1.4%	7.9%	90.6%	
Non-Hispanic Black	566	0.2%	0.5%	9.7%	89.6%	
Other Races Including Multi-Racial	409	0.0%	1.0%	10.8%	88.3%	
**Household income**						
Below Poverty Line	843	0.1%	1.2%	8.8%	89.9%	0.822
Near Poverty	112	0.0%	1.8%	8.0%	90.2%	
Low-Income	419	0.0%	1.0%	7.6%	91.4%	
Middle-Income	320	0.3%	0.9%	10.0%	88.8%	
High-Income	1070	0.1%	0.8%	8.8%	90.3%	
**Education level**						
Less than High School	568	0.0%	0.7%	6.5%	92.8%	0.050 *
High School Level	620	0.2%	1.3%	10.0%	88.5%	
AA or College Degree	1495	0.1%	1.0%	8.8%	90.0%	
**Mental health**						
Not at all	2316	0.1%	0.9%	8.9%	90.1%	0.686
Several days	288	0.0%	2.4%	6.9%	90.6%	
More than half the days	70	0.0%	0.0%	11.4%	88.6%	
Nearly every day	90	0.0%	0.0%	7.9%	92.1%	
**Occupation**						
Working	1470	0.0%	1.4%	8.2%	0.0%	0.343
Not Working	1294	0.2%	0.6%	9.3%	0.2%	
**Marital status**						
Married	2157	0.00%	0.00%	6.60%	93.40%	0.001 *
Not Married	607	0.10%	1.30%	9.30%	89.20%	
**Alcohol consumption status**						
Non-drinker	1770	0.2%	1.1%	8.3%	90.4%	0.311
Drinker	994	0.0%	0.8%	9.5%	89.7%	
**Weight status (Based on BMI)**						
Underweight	0.0%	1.0%	7.7%	91.3%	0.0%	0.524
Normal	0.0%	0.9%	8.7%	90.4%	0.0%	
Overweight	0.3%	1.4%	9.2%	89.0%	0.3%	
Obese	0.1%	0.7%	9.5%	89.6%	0.1%	
**Diabetes**						
Present	277	0.7%	0.7%	8.3%	90.3%	0.529
Not present	2487	0.0%	1.0%	8.8%	90.1%	

^a^ Weighted row percentage.

* Statistically significant at 0.05.

**Table 3.** Distribution of Periodontitis per Dental Visits, Insurance Coverage, Occupation, and, Marital Status Among Study Participants (n=2764).

	Total (n)	Severe periodontitis %	Moderate periodontitis %	Mild periodontitis%	No periodontitis %	*P*-value
**Time of most recent dental visit**						
Less than 1 year	1754	0.1%	1.2%	8.6%	90.1%	0.998
1-2 years	274	0.4%	0.4%	9.1%	90.1%	
More than 2 years	736	0.1%	0.8%	8.8%	90.2%	
**Health Insurance**						
Yes	2255	0.1%	0.9%	7.3%	91.7%	0.001 ^*^
No	509	0.0%	1.4%	15.1%	83.5%	

^a^ Weighted row percentage.

* Statistically significant at 0.05.

Unadjusted odds ratios (OR) were computed through logistic regression, controlling for all confounders, and are presented with a 95% confidence interval (CI) in Table 4. The logistic regression analysis indicated that the odds of periodontal disease were higher among those who reported feeling bad about themselves for more than half of the day compared to those who reported not feeling that way at all (OR 1.170, 95% CI 0.533-2.474). However, this association was not statistically significant (p>0.05).

**Table 4.** Logistic regression analysis of the effects of mental health on periodontitis.

Variable	OR (95% CI)
**Mental health**	
Not at all	Ref
Several days	0.938 (0.617-1.426)
More than half the days	1.170 (0.533-2.473)
Nearly every day	0.765 (0.349-1.674)

No Periodontitis=0 vs. Periodontitis (all three)=1.

## Discussion

The primary objective of this study was to examine the relationship between mental health (self-perception) and periodontal disease. The study findings revealed no discernible difference in the prevalence of periodontitis between individuals with mental health issues and those without. However, it is noteworthy that the odds of periodontal disease were somewhat higher among those who reported feeling bad about themselves for more than half of the day, though this difference did not reach statistical significance. Additionally, male participants exhibited a higher likelihood of periodontal disease compared to female participants. Furthermore, lacking insurance coverage was significantly associated with the prevalence of periodontitis.

The study findings presented here cannot be directly compared with other studies to date due to variations in the measures used to assess stress and psychosocial conditions. Nevertheless, common factors across these studies include stress as a factor and the outcome variable, which is periodontal disease.

Monteiro da Silva et al. (1998) found no significant difference between psychosocial stress and periodontal disease. ^27^ Similarly, Solis et al. (2004) reported no association between depression, hopelessness, psychiatric symptoms, and established or severe periodontitis. ^28^ Castro et al. (2006) also concluded that there was no significant association between periodontitis and the analyzed psychosocial factors. ^29^ The present study aligns with the findings of Shende et al. (2016), where the oral hygiene and periodontal status of individuals with mild anxiety and depression were not different from those without anxiety and depression. ^30^ Thus, this study does not demonstrate stress as a causative factor for periodontal disease, despite observing a difference in the periodontal status of individuals with or without normal mental health, which was not statistically significant.

The current study’s results are consistent with Genco et al.’s (1998) findings, indicating that various measures of stress, including life events and daily strains, were not correlated with periodontal disease. Only financial strain showed a significant association with greater attachment loss and alveolar bone loss after adjusting for variables such as age and smoking. Participants with higher financial strain and those with depression had a significantly higher risk for periodontal disease. ^11^

One intriguing observation in the present study was the lower prevalence of periodontal disease and related findings in individuals with dental insurance compared to those without insurance. The possession of dental insurance appears to contribute to a sense of mental well-being, potentially reducing financial-related stress and mitigating the negative impact on periodontal disease. This aligns with the findings of Genco et al. (1998), who demonstrated a correlation between financial strain and poor periodontal health. ^11^ Similarly, a study exploring the relationship between mental health and oral health in older individuals in Australia highlighted that the availability of free treatment or provision of dental insurance offers a sense of comfort in daily life, potentially shielding against mental health issues. ^31^ Ng and Leung (2006) also support this perspective, suggesting that individuals with higher mean clinical attachment level values tended to have higher scores on job and financial strain scales compared to periodontally healthy individuals. ^32^

The findings of the present study diverge from those reported by Mahendra et al. (2011), Reshma et al. (2013), Vyas et al. (2018), and various other animal and human studies. ^23^^,^^24^^,^^33^ These studies employed diverse stress measures, including cortisol levels and other stress assessment methods. Discrepancies between the present study’s findings and those of the aforementioned studies could arise from differences in methodology, the type of assessment employed, considered co-variables, and the demographic characteristics of the study participants. Many studies focused on specific groups exposed to stress, such as police personnel or older individuals, whereas our study encompassed a broader sample, considering individuals from various backgrounds in relation to mental health. The diverse nature of the study population may contribute to the variations observed in the results.

The current investigation has notable strengths, including its reliance on data from a large-scale population-based survey with a robust sampling and weighting system. This survey encompassed a diverse range of participants, including both females and males, various age groups, individuals with different socioeconomic statuses, and those with and without insurance. The inclusion of these variables, which are not often considered collectively in previous studies, enhances the reliability of the associations found within this research. However, there are limitations to consider. Firstly, mental health status was assessed based on self-reported answers from the participants, which may introduce subjectivity and potential bias into the study outcomes. The broad and complex nature of assessing mental health on a single-point measure or limited parameters may not fully capture the true mental health status of individuals. Additionally, the cross-sectional design of the study limits the ability to infer causality in the observed associations. The study does not confirm the outcome, and the link between stress-associated odds of periodontal disease and behavioral or pathophysiological changes remains to be determined.

Moreover, the study acknowledges the potential bidirectional relationship between oral health concerns and mental health status. For instance, the appearance of teeth, mouth, or dentures, as well as problems with them, might impact social interaction, potentially contributing to mental health issues such as feelings of hopelessness and depression. Controlling for these variables in a study investigating the relationship between mental health status and periodontal disease becomes challenging, and the true measured outcome may be affected.

To enhance future research, prospective studies with a focus on biochemical and physiological mechanisms by which psychosocial stress contributes to periodontal destruction are needed. These studies could establish the biological rationale for the observed relationship. Furthermore, refining the definition of stress and employing a standardized protocol to assess stress and associated factors would contribute to clearer comparisons of periodontal status among individuals.

## Conclusion

In conclusion, within the constraints of this study, it can be deduced that there is no statistically significant difference in the periodontal status between individuals with compromised mental health and those without such issues. Our findings suggest trends in periodontal disease prevalence linked to mental health, marital status, and access to health insurance. However, the absence of statistically significant findings calls for caution in interpreting these relationships. We recommend that future studies further investigate these potential associations to provide a clearer understanding.

## Ethics and consent

The protocols for collecting oral health data in the NHANES 2011–2012 and NHANES 2013–2014 cycles received approval from the Centers for Disease Control and Prevention National Center for Health Statistics Research Ethics Review Board. All survey participants provided written informed consent before publishing their information. https://www.cdc.gov/nchs/nhanes/irba98.htm

## Data Availability

Aljoghaiman, Eman (2024). The Relationship Between Mental Health and Periodontal Disease: Insights from NHANES Data. figshare. Dataset. https://doi.org/10.6084/m9.figshare.25662735 ^34^ Data are available under the terms of the Creative Commons Attribution 4.0 International license (CC-BY 4.0)
