# Supplementary material for: The Relationship Between Mental Health and Periodontal Disease: Insights from NHANES Data
Source: F1000Res. 2025 Oct 31;13:709. Originally published 2024 Jun 27. [Version 3] doi: 10.12688/f1000research.150837.3 (PMC11781522; doi:10.12688/f1000research.150837.3)
Supplement: Peer review history

# Reviewer report: Reviewer response for version 3

**DOI:** 10.5256/f1000research.190535.r428868

**Published:** 2025-11-08

**Authors:** Morenike Folayan

**Affiliations:** 1 Obafemi Awolowo University, Ife, Osun, Nigeria

Approved

Is the work clearly and accurately presented and does it cite the current literature?

Partly

If applicable, is the statistical analysis and its interpretation appropriate?

No

Are all the source data underlying the results available to ensure full reproducibility?

Yes

Is the study design appropriate and is the work technically sound?

Partly

Are the conclusions drawn adequately supported by the results?

Partly

Are sufficient details of methods and analysis provided to allow replication by others?

No

Reviewer Expertise:

NA

I confirm that I have read this submission and believe that I have an appropriate level of expertise to confirm that it is of an acceptable scientific standard.

# Reviewer report: Reviewer response for version 2

**DOI:** 10.5256/f1000research.173275.r346478

**Published:** 2024-12-17

**Authors:** Nancy Ajwa

**Affiliations:** 1 College of Dentistry, Riyadh Elem University, Riyadh, Saudi Arabia

Thank you, authors, for the revised version.

 The authors have addressed previous comments very well. The newly modified introduction is in good shape now. In the methodology section, please mention your study period as well. The tables are now well-constructed. The discussion becomes more insightful with the limitations added. The conclusion aligns well with the results and objectives.

Is the work clearly and accurately presented and does it cite the current literature?

Yes

If applicable, is the statistical analysis and its interpretation appropriate?

Yes

Are all the source data underlying the results available to ensure full reproducibility?

Yes

Is the study design appropriate and is the work technically sound?

Yes

Are the conclusions drawn adequately supported by the results?

Yes

Are sufficient details of methods and analysis provided to allow replication by others?

Yes

Reviewer Expertise:

Public Health, Dentistry, Education, Biostatistics, Periodontology, Orthodontology,

I confirm that I have read this submission and believe that I have an appropriate level of expertise to confirm that it is of an acceptable scientific standard.

# Reviewer report: Reviewer response for version 2

**DOI:** 10.5256/f1000research.173275.r339002

**Published:** 2024-12-17

**Authors:** Morenike Folayan

**Affiliations:** 1 Obafemi Awolowo University, Ife, Osun, Nigeria

**Please find unaddressed comments**

1. **Comment: **What demographic variables were included in the analysis. Abstract is 300 words and the authors have less than that. Please include the details.
2. **Comment: **How was mental health measured for this study in the methodology -
3. You wrote in response to my comment - **Response: **Our findings suggest trends in periodontal disease prevalence linked to mental health, marital status, and access to health insurance. However, the absence of statistically significant findings calls for caution in interpreting these relationships. We recommend that future studies further investigate these potential associations to provide a clearer understanding. ***PLEASE NOTE, A CROSS SECTIONAL STUCY CANNOT ESTABLISH TRENDS. PLEASE REVISE THE LANGUAGE OF THE CONCLUDING STATEMENTS.***
4. **Response: **We listed them as covariates…. ***YET THE AUTHORS IN THIS STATEMENT, SEEM TO SUGGEST THEY ARE CONFOUNDERS*** - By meticulously examining and accounting for these covariates, our objective is to obtain results that reflect the true relationship between the exposure and outcome, minimizing the confounding effects of other variables.
5. **Comment: **What informed the age categorisation? There is no justification for the age categorisation nor is the interpretation and application of the findings meaningful based on this age categorisation. Secondly, since this data is large and likely not be skewed, it is best to treat age as a continuous variable. – ***PLEASE TREAT AGE AS A CONTINUOUS VARIABLE IN YOUR ANALYSIS***
6. **Comment: **Please how were these variables measured - education, socioeconomic status, poverty/income ratio, marital status, BMI, HbA1C, dental insurance coverage, dental visit frequency, and mental health assessment – ***PLEASE INCLUDE THE INFORMATION IN THE METHODS SECTION WITH REFERENCES WHERE APPROPRIATE***
7. **Comment: **Please how was the data on poverty indices collected before being categorized into low, middle, and high? - ***PLEASE INCLUDE THE INFORMATION IN THE METHODS SECTION WITH REFERENCES WHERE APPROPRIATE***
8. **Comment: **Please did the authors check for collinearity between the variables such as education, socioeconomic status, poverty/income ratio? - ***PLEASE INCLUDE THE INFORMATION IN THE METHODS SECTION ON HOW COLLINEARITY WAS ADDRESSED***
9. **Comment: **The authors wrote - ‘Additionally, a logistic regression model was utilized to assess the influence of mental health on periodontal disease’. This was a cross-sectional study and so the data cannot give information on influence. All it can suggest are risk indicators based on the results of the associations. – ***PLEASE CORRECT THE REFERENCE TO INFLUENCE AND TREND THROUGHOUT THE MANUSCRIPT. THE STUDY DESIGN CANNOT GENERATE THIS INFORMATION.***
10. **Comment: **Please do not round up the figures to report a quarter etc. Please be precise with the results. You can round up in the discussion.
11. **Comment: **Table 4 is not clear – what were the confounding variables adjusted for in this table. ***ONCE AGAIN. I AM BACK ON THE NON-CLARITY ON THE COVARIATES AS CONFOUNDERS. IF YOU TREATED THE COVARIATES AS CONFOUNDERS THEN PLEASE STATE THEM AS CONFOUNDERS AS CONFOUNDERS AND COVARIATES ARE NOT THE SAME. IF THE MODEL WAS ADJUSTED FOR CONFOUNDERS THEN REPORT AOR AND NOT OR.***

**NEW COMMENTS**

1. **Comment: **This study aims to investigate the relationship between mental health and periodontal disease – what relationship – association, trend, correlation? Be specific?
2. TABLE 2: THE variables ARE NOT predictors BUT risk indicators As this was A cross Sectional study. Please correct
3. Throughout the document, please use small letter ‘P’articipants for participants when used within sentences.
4. Throughout the document, please ezclude in-references to years like (1998)

Is the work clearly and accurately presented and does it cite the current literature?

Partly

If applicable, is the statistical analysis and its interpretation appropriate?

No

Are all the source data underlying the results available to ensure full reproducibility?

Yes

Is the study design appropriate and is the work technically sound?

Partly

Are the conclusions drawn adequately supported by the results?

Partly

Are sufficient details of methods and analysis provided to allow replication by others?

No

Reviewer Expertise:

epidemiology and oral health

I confirm that I have read this submission and believe that I have an appropriate level of expertise to confirm that it is of an acceptable scientific standard, however I have significant reservations, as outlined above.

# Reviewer report: Reviewer response for version 2

**DOI:** 10.5256/f1000research.173275.r339003

**Published:** 2024-11-20

**Authors:** Junaid Ahmed

**Affiliations:** 1 Department of Oral Medicine & Radiology, Manipal College of Dental Sciences, Mangalore, Karnataka, India

The modifications in the manuscript are according to the suggestions and is satisfactory

Is the work clearly and accurately presented and does it cite the current literature?

Partly

If applicable, is the statistical analysis and its interpretation appropriate?

I cannot comment. A qualified statistician is required.

Are all the source data underlying the results available to ensure full reproducibility?

Partly

Is the study design appropriate and is the work technically sound?

Partly

Are the conclusions drawn adequately supported by the results?

Partly

Are sufficient details of methods and analysis provided to allow replication by others?

Partly

Reviewer Expertise:

Forensic Odontology,TMJ disorders,Oral Cancer,3D Radiology

I confirm that I have read this submission and believe that I have an appropriate level of expertise to confirm that it is of an acceptable scientific standard.

# Reviewer report: Reviewer response for version 1

**DOI:** 10.5256/f1000research.165439.r317918

**Published:** 2024-09-16

**Authors:** Junaid Ahmed

**Affiliations:** 1 Department of Oral Medicine & Radiology, Manipal College of Dental Sciences, Mangalore, Karnataka, India

1.The inclusion & exclusion criteria needs to be listed more elaborately

 2.The authors have not mentioned their source of demographic data including the country from where the data is sourced.

 3..Was the NHANES data previously published by the government?.The authors need to clarify.

 4.Have the authors taken the necessary permission to publish the data from the competent sources?

 5.The study setting in which the objectives of the present research is mentioned is not clear in the manuscript.

 6.The authors need to mention the sample size of the data and whether any methodology was utilized for sample size estimation.

 7.The self reported mental health status is a big limitation in the present study and any direct relationship between mental health & periodontal disease cannot be inferred from the present study.

 8.Quality of written english needs to be improved and a few incoherent sentences needs to be rephrased.

 9.A few comments have been highlighted in the attached manuscript

Is the work clearly and accurately presented and does it cite the current literature?

Partly

If applicable, is the statistical analysis and its interpretation appropriate?

I cannot comment. A qualified statistician is required.

Are all the source data underlying the results available to ensure full reproducibility?

Partly

Is the study design appropriate and is the work technically sound?

Partly

Are the conclusions drawn adequately supported by the results?

Partly

Are sufficient details of methods and analysis provided to allow replication by others?

Partly

Reviewer Expertise:

Forensic Odontology,TMJ disorders,Oral Cancer,3D Radiology

I confirm that I have read this submission and believe that I have an appropriate level of expertise to confirm that it is of an acceptable scientific standard, however I have significant reservations, as outlined above.

# Response

**Published:** 2024-10-09

**Authors:** Eman AlJoghaiman

**Affiliations:** Imam Abdulrahman Bin Faisal University, Dammam, Eastern Province, Saudi Arabia

Dear Reviewer,

 We would like to express our sincere gratitude for your insightful review and valuable suggestions regarding our manuscript. We have carefully revised the manuscript and provided detailed responses to your comments, which are included alongside the revised version.

 Thank you very much for your time and consideration.

 Dr. Eman

# Response

**Published:** 2024-10-09

**Authors:** Eman AlJoghaiman

**Affiliations:** Imam Abdulrahman Bin Faisal University, Dammam, Eastern Province, Saudi Arabia

Dear Reviewer,

 We would like to express our sincere gratitude for your insightful review and valuable suggestions regarding our manuscript. We have carefully revised the manuscript and provided detailed responses below to your comments one by one. Please let us know if you have any further questions.

 Thank you for your time.

**Reviewer comment 1: **The inclusion & exclusion criteria needs to be listed more elaborately

**Author response:** Added in the attached manuscript. Thank you

**Reviewer comment 2: **The authors have not mentioned their source of demographic data including the country from where the data is sourced.

**Author response:** The National Health and Nutrition Examination Survey (NHANES) is a comprehensive cross-sectional survey conducted in the United States, designed to offer a nationally representative overview of non-institutionalized individuals residing in households.

**Reviewer comment 3: **Was the NHANES data previously published by the government?. The authors need to clarify.

**Author response:** Yes, it is published and publicly available for other to re-use the data. The source of the data is provided in the method section.  Thank you

**Reviewer comment 4: **Have the authors taken the necessary permission to publish the data from the competent sources?

**Author response: **Yes, the data is available freely. Thank you

**Reviewer comment 5: **The study setting in which the objective of the present research is mentioned is not clear in the manuscript.

**Author response:** Can you please specify what exactly should be added?

**Reviewer comment 6: **The authors need to mention the sample size of the data and whether any methodology was utilized for sample size estimation.

**Author response:** No sample size was calculated because authors utilized whole data available in the website. Thank you

**Reviewer comment 7: **The self reported mental health status is a big limitation in the present study and any direct relationship between mental health & periodontal disease cannot be inferred from the present study.

**Author response:** Noted thank you

**Reviewer comment 8: **Quality of written English needs to be improved and a few incoherent sentences needs to be rephrased.

**Author response:** Thank you for the comment, we have done English proofread and rephrased the sentences for more readability.

**Reviewer comment 9: **A few comments have been highlighted in the attached manuscript

**Author response:**  Thank you for the comments.

# Reviewer report: Reviewer response for version 1

**DOI:** 10.5256/f1000research.165439.r306426

**Published:** 2024-08-13

**Authors:** Morenike Folayan

**Affiliations:** 1 Obafemi Awolowo University, Ife, Osun, Nigeria

The Relationship Between Mental Health and Periodontal Disease: Insights from NHANES Data

 Thanks for this very important study generated from a secondary analysis of a large database. Please find below suggestions to strengthen the manuscript

 Abstract

- Please include clearly that this was a secondary data analysis of the NHANEs data
- Include information on the period the data was collected
- Please provide details on the covariate variables extracted
- What kind of analysis was conducted with the Rao-Scott chi-squared test. Please avoid making readers assume what was done.
- What demographic variables were included in the analysis
- What disease status is been referred too?
- How was mental health measured for this study?
- What is the sample size for this study? This information should be included in the result section
- For the report, please report the descriptive variables before the inferential analysis reports.
- Authors noted a significant difference in periodontal status by marital status and by health insurance status but there is no report on the CI or the p value of these observations. There is also no clarity about how the results of these tests of associations answer the study objectives as the objective had Mental health as the dependent variable.
- Is ‘feeling bad about themselves for more than half of the day’ the measure of mental health for this study?
- The conclusion that ‘The findings underscore potential associations between mental health, marital status, and access to health insurance with periodontal disease’ is not valid based on this study results.

INTRODUCTION

- A comprehensive introduction
- The introduction is missing on the conceptual framework that suggest that first suggests that self-perception is a mental health issue, and that there are suggestive links between self-perception (about what) and periodontal diseases. Please address this concisely in the introduction.

METHODs

- Please provide explicit details on how mental health was measured.
- Please be explicit about the periodontal index used for this study.
- I think what is listed as covariates are defined as confounders. These needs to be clarified please.
- What informed the age categorisation? There is no justification for the age categorisation nor is the interpretation and application of the findings meaningful based on this age categorisation. Secondly, since this data is large and likely not be skewed, it is best to treat age as a continuous variable.
- Please how were these variables measured - education, socioeconomic status, poverty/income ratio, marital status, BMI, HbA1C, dental insurance coverage, dental visit frequency, and mental health assessment
- Please how was the data on poverty indices collected before being categorized into low, middle, and high?
- Please did the authors check for collinearity between the variables such as education, socioeconomic status, poverty/income ratio?
- This statement is not clear: ‘To analyze the demographics and disease status of the study population, the Rao-Scott chi-squared test was employed, taking into consideration the survey design complexities’. The Rao-Scott chi-squared test is a test of association. Not sure how this can be an analytic tool for ‘the demographics and disease status of the study population’
- The authors wrote - ‘Additionally, a logistic regression model was utilized to assess the influence of mental health on periodontal disease’. This was a cross sectional study and so the data cannot give information on influence. All it can suggest are risk indicators based on the results of the associations.

Results

- Please for the results, write the figures and the percentage in brackets
- Please do not round up the figures to report a quarter etc. Please be precise with the results. You can round up in the discussion.
- Please can you report the study participants as participants rather than subjects
- The asterisks in the Table 2 suggests that Rao-Scott chi-square test as not applied to all variables
- Table 2 also suggests that the variables listed in the methodology were treated as co-variates and not confounders. Please be clear with your methodology and the use of terminologies.
- The test of association cannot measure effect. Please the title to the section is misleading
- Please in the narrative on tables 2 and 3, please include information on the determinants of the report of significant associations
- Please be specific on the percentage and type of periodontal disease referred too. The table did not report on ‘some forms’ of periodontal diseases
- Table 4 is not clear – what were the confounding variables adjusted for in this table.

In view of the non-clarity of the methodology and results, I am unable to review the discussion until this is clarified. The authors are encouraged to use the TROBE guidelines to guide the review and the structuring of the paper.

Is the work clearly and accurately presented and does it cite the current literature?

Partly

If applicable, is the statistical analysis and its interpretation appropriate?

No

Are all the source data underlying the results available to ensure full reproducibility?

Yes

Is the study design appropriate and is the work technically sound?

Partly

Are the conclusions drawn adequately supported by the results?

Partly

Are sufficient details of methods and analysis provided to allow replication by others?

No

Reviewer Expertise:

epidemiology and oral health

I confirm that I have read this submission and believe that I have an appropriate level of expertise to state that I do not consider it to be of an acceptable scientific standard, for reasons outlined above.

# Response

**Published:** 2024-10-09

**Authors:** Eman AlJoghaiman

**Affiliations:** Imam Abdulrahman Bin Faisal University, Dammam, Eastern Province, Saudi Arabia

Dear Reviewer,

 We would like to express our sincere gratitude for your insightful review and valuable suggestions regarding our manuscript. We have carefully revised the manuscript and provided detailed responses below to your comments one by one. Please let us know if you have any further questions.

 Thank you for your time.

**Reviewer 1:**

 Thanks for this very important study generated from a secondary analysis of a large database. Please find below suggestions to strengthen the manuscript.

**Comment:** Abstract: Please include clearly that this was a secondary data analysis of the NHANEs data

 - **Response**: Added

**Comment: **Include information on the period the data was collected

 - **Response**: Added

**Comment: **Please provide details on the covariate variables extracted

 - **Response:** Added

**Comment: **What kind of analysis was conducted with the Rao-Scott chi-squared test. Please avoid making readers assume what was done.

 - **Response:** It is a chi-square test used for association. Thank you

**Comment: **What demographic variables were included in the analysis

 - **Response:** All demographic variables included are present in the material method section below because listing them here will be difficult because of word limitation for abstract.

**Comment: **What disease status is been referred too?

 - **Response:** Periodontal disease

**Comment: **How was mental health measured for this study?

 - **Response: **Added in the material method section

**Comment: **What is the sample size for this study? This information should be included in the result section

 - **Response: **Added

**Comment: **For the report, please report the descriptive variables before the inferential analysis reports.

 - **Response:** Corrected

**Comment: **Authors noted a significant difference in periodontal status by marital status and by health insurance status but there is no report on the CI or the p value of these observations. There is also no clarity about how the results of these tests of associations answer the study objectives as the objective had Mental health as the dependent variable.

 - **Response:** Periodontal status is the dependent variable, and details are added in the result section. Thank you

 Is ‘feeling bad about themselves for more than half of the day’ the measure of mental health for this study?

 - **Response:** Yes

**Comment: **The conclusion that ‘The findings underscore potential associations between mental health, marital status, and access to health insurance with periodontal disease’ is not valid based on this study results.

 - **Response: **Our findings suggest trends in periodontal disease prevalence linked to mental health, marital status, and access to health insurance. However, the absence of statistically significant findings calls for caution in interpreting these relationships. We recommend that future studies further investigate these potential associations to provide a clearer understanding

**INTRODUCTION**

**Comment: **A comprehensive introduction

 - **Response:** Modified. Thank you

**Comment: **The introduction is missing on the conceptual framework that suggest that first suggests that self-perception is a mental health issue, and that there are suggestive links between self-perception (about what) and periodontal diseases. Please address this concisely in the introduction.

 - **Response:** Based on the reviewer’s comment regarding the ⁠The introduction is missing on the conceptual framework authors have revise the introduction. Thank you

**METHOD**

**Comment: **Please provide explicit details on how mental health was measured.

 - **Response:** Added

**Comment: **Please be explicit about the periodontal index used for this study.

 - **Response: **Added

**Comment: **I think what is listed as covariates are defined as confounders. These needs to be clarified please.

 - **Response: **We listed them as covariates….

**Comment: **What informed the age categorisation? There is no justification for the age categorisation nor is the interpretation and application of the findings meaningful based on this age categorisation. Secondly, since this data is large and likely not be skewed, it is best to treat age as a continuous variable.

 - **Response:** Age categorization was done to check the variability in the different age groups.

**Comment: **Please how were these variables measured - education, socioeconomic status, poverty/income ratio, marital status, BMI, HbA1C, dental insurance coverage, dental visit frequency, and mental health assessment

 - **Response:** Thank you for the comment, authors have listed all categories about the measurements in table 1.

**Comment: **Please how was the data on poverty indices collected before being categorized into low, middle, and high?

 - **Response:** Based on the poverty income ratios, categories were created to represent different socioeconomic status groups. Here's a common approach:

 Below Poverty Line: Participants with a poverty income ratio less than 1.0 are considered below the poverty line.

 Near Poverty: Participants with a poverty income ratio between 1.0 and 1.25 are often categorized as near-poverty.

 Low-Income: Participants with a poverty income ratio between 1.25 and 2.0 are typically categorized as low-income.

 Middle-Income: Participants with a poverty income ratio between 2.0 and 3.0 are considered middle-income.

 High-Income: Participants with a poverty income ratio greater than 3.0 are categorized as high-income.

**Comment: **Please did the authors check for collinearity between the variables such as education, socioeconomic status, poverty/income ratio?

 - **Response:** The authors appreciate your feedback and will consider using collinearity diagnostics in future studies to improve the rigor of our findings. Our investigation focused on ensuring that our regression models were robust and reliable. While we did not directly collect collinearity statistics, we were aware of the possibility of multicollinearity when selecting and incorporating variables in our models.

**Comment: **This statement is not clear: ‘To analyze the demographics and disease status of the study population, the Rao-Scott chi-squared test was employed, taking into consideration the survey design complexities’. The Rao-Scott chi-squared test is a test of association. Not sure how this can be an analytic tool for ‘the demographics and disease status of the study population’

 - **Response:** The lines are Rephrased.

**Comment: **The authors wrote - ‘Additionally, a logistic regression model was utilized to assess the influence of mental health on periodontal disease’. This was a cross-sectional study and so the data cannot give information on influence. All it can suggest are risk indicators based on the results of the associations.

 - **Response:** Thank you for your suggestion.

**Results**

**Comment: **Please for the results, write the figures and the percentage in brackets

 - **Response:** Done

**Comment: **Please do not round up the figures to report a quarter etc. Please be precise with the results. You can round up in the discussion.

 - **Response:** Thank you

**Comment: **Please can you report the study participants as participants rather than subjects

 - **Response:** Done, Thank You

**Comment: **The asterisks in the Table 2 suggests that Rao-Scott chi-square test as not applied to all variables

 - **Response: **Corrected, Thank you

**Comment: **Table 2 also suggests that the variables listed in the methodology were treated as co-variates and not confounders. Please be clear with your methodology and the use of terminologies.

 - **Response:** Corrected

**Comment: **The test of association cannot measure effect. Please the title to the section is misleading

 - **Response:** Corrected

**Comment: **Please in the narrative on tables 2 and 3, please include information on the determinants of the report of significant associations

 - **Response: **Corrected

**Comment: **Please be specific on the percentage and type of periodontal disease referred too. The table did not report on ‘some forms’ of periodontal diseases

 - **Response: **Corrected

**Comment: **Table 4 is not clear – what were the confounding variables adjusted for in this table.

 - **Response: **All demographical characteristics.

**Comment: **In view of the non-clarity of the methodology and results, I am unable to review the discussion until this is clarified. The authors are encouraged to use the TROBE guidelines to guide the review and the structuring of the paper.

 - **Response:** Thank you for your valuable review
